# Characterisation of Grains and Flour Fractions from Field Grown Transgenic Oil-Accumulating Wheat Expressing Oat *WRI1*

**DOI:** 10.3390/plants11070889

**Published:** 2022-03-26

**Authors:** Per Snell, Mark Wilkinson, Gavin J. Taylor, Stephen Hall, Shrikant Sharma, Nick Sirijovski, Mats Hansson, Peter R. Shewry, Per Hofvander, Åsa Grimberg

**Affiliations:** 1 Department of Plant Breeding, Swedish University of Agricultural Sciences, 23456 Alnarp, Sweden; per.snell@dlf.com (P.S.); shrikant.sharma@slu.se (S.S.); per.hofvander@slu.se (P.H.); 2 DLF Beet Seed AB, 26191 Landskrona, Sweden; 3 Department of Plant Sciences, Rothamsted Research, Harpenden AL5 2JQ, UK; mark.wilkinson@rothamsted.ac.uk (M.W.); peter.shewry@rothamsted.ac.uk (P.R.S.); 4 Department of Biology, Lund University, 22362 Lund, Sweden; gavin.taylor@igdore.org (G.J.T.); mats.hansson@biol.lu.se (M.H.); 5 Institute for Globally Distributed Open Research and Education, Ribeirão Preto 14091-310, Brazil; 6 Division of Solid Mechanics, Lund University, 22363 Lund, Sweden; stephen.hall@solid.lth.se; 7 Lund Institute of Advanced Neutron and X-ray Science (LINXS), 22370 Lund, Sweden; 8 Department of Chemistry, Pure and Applied Biochemistry, ScanOats, Lund University, 22100 Lund, Sweden; nick.sirijovski@tbiokem.lth.se

**Keywords:** aleurone cells, *Triticum aestivum*, carbon allocation, cereal endosperm, oil biosynthesis, transcription factor, triacylglycerol, wheat flour, WRINKLED1

## Abstract

Wheat (*Triticum aestivum* L.) is one of the major staple crops in the world and is used to prepare a range of foods. The development of new varieties with wider variation in grain composition could broaden their use. We characterized grains and flours from oil-accumulating transgenic wheat expressing the oat (*Avena sativa* L.) endosperm *WRINKLED1* (*AsWRI1*) grown under field conditions. Lipid and starch accumulation was determined in developing caryopses of *AsWRI1*-wheat and X-ray microtomography was used to study grain morphology. The developing caryopses of *AsWRI1*-wheat grains had increased triacylglycerol content and decreased starch content compared to the control. Mature *AsWRI1*-wheat grains also had reduced weight, were wrinkled and had a shrunken endosperm and X-ray tomography revealed that the proportion of endosperm was decreased while that of the aleurone was increased. Grains were milled to produce two white flours and one bran fraction. Mineral and lipid analyses showed that the flour fractions from the *AsWRI1-*wheat were contaminated with bran, due to the effects of the changed morphology on milling. This study gives a detailed analysis of grains from field grown transgenic wheat that expresses a gene that plays a central regulatory role in carbon allocation and significantly affects grain composition.

## 1. Introduction

Wheat (*Triticum aestivum* L.) is one of the major food crops in the world and accounts for approximately 25% of total cereal production (www.fao.org/faostat, 2 February 2022). It is cultivated in temperate zones all over the world and is not only a rich source of carbohydrates but also contains significant amounts of proteins and fiber plus minor compounds, such as lipids, vitamins, minerals and phytochemicals [1]. The different components are spatially distributed between and within the different tissues of the cereal grain, which has implications for end uses, notably milling to produce white flour for breadmaking [2]. Wheat grains are used as raw material for a range of food items, and the development of new wheat varieties with wider variation in grain composition and properties could broaden their use. However, to achieve this goal, we require a more detailed understanding of the genetic regulation of nutrient accumulation in different wheat grain tissues. Wheat varieties with increased lipid content are not readily available but could be of interest for applications, such as bread baking, where typically vegetable oil is added to the process [3]. It is, therefore, interesting to explore how increased oil content of wheat grains affects grain development, as well as the nutritional and processing properties of high-oil wheat flour. A more diverse portfolio of wheat varieties with different grain compositions and properties could also provide opportunities for wider uses. This has been demonstrated for commercially available low and high oil varieties of oats (*Avena sativa* L.) which are typically used for food and feed purposes, respectively. In addition, there is increased interest in using different oat lipids in high-value food and pharmaceutical applications [4].

Wheat grains consist of several tissues and milling is used to separate the starchy endosperm (83% by dry weight (dw)) as white flour from the embryo (3% by dw), aleurone and outer grain layers (14% by dw) which together form the bran [5]. The starchy endosperm, and hence white flour, contains 75–85% starch and 10–15% protein by dw. Oil (triacylglycerol; TAG) is deposited mainly in the embryo and aleurone layer with smaller amounts in the starchy endosperm cells, giving an oil content of only 3% of the total grain dw [6]. The development of high-oil maize (*Zea mays* L.) cultivars has been shown to result from increased size and oil content of the embryo [7] and the oil content in rice (*Oryza sativa* L.) grains has been shown to be positively correlated to the thickness of aleurone cells [8]. However, oats are unique among the cereals in also storing significant amounts of oil in the starchy endosperm, and the oil level can reach up to 18% by total grain dw [9,10,11,12]. These examples show that plant breeding can target different grain tissues to achieve an increased oil content.

WRINKLED1 (WRI1) is a transcription factor known to regulate the allocation of carbon into fatty acid synthesis in plant storage tissues and is, therefore, an attractive target for biotechnological applications aiming at increased oil content. WRI1 was first discovered and characterized in Arabidopsis [13,14] and has since been identified in the storage tissue of several different plant species [15,16,17,18,19,20], including the oat endosperm [21,22,23]. In our recent work [24] we explored if it was possible to modify carbon allocation to increase the oil content in the starchy endosperm of wheat by endosperm-specific expression of the oat endosperm *WRI1* (*AsWRI1*). Our results, from experiments performed under greenhouse conditions, showed that the grain composition of this genetically modified wheat (*AsWRI1*-wheat) was significantly different compared to the control and resulted in increased oil content, from 0.7% to 6.4% by grain dw, accompanied by a large decrease in starch content and an increase in sugars [24]. Morphological changes were also observed in the oil-accumulating wheat, which had wrinkled grains with a greatly reduced starchy endosperm, and with elongated peripheral aleurone cells that, at some positions, were present in multiple layers (the aleurone layer usually being a single cell thick in wheat [25]). We hypothesize that the trait with increased oil content and changed seed morphology in *AsWRI1*-wheat grains can also be manifested under field conditions, yielding a wheat flour with different nutritional quality and higher nutritional density (from the additional oil) as compared to flour prepared from grains of control plants.

In this work, we determine nutrient accumulation in the developing endosperm of the oil-accumulating *AsWRI1*-wheat under open field conditions and analyze the lipid and mineral compositions in different flour fractions by milling. We also explore the 3D morphological changes occurring in *AsWRI1*-wheat grains by high-resolution X-ray microtomography. The study provides a detailed analysis of the field grown grain of transgenic wheat, which expresses a gene that plays a central regulatory role in carbon allocation and significantly affects grain composition and quality.

## 2. Results

### 2.1. Field Trial of Genetically Modified Wheat in Sweden

**Figure 1:**
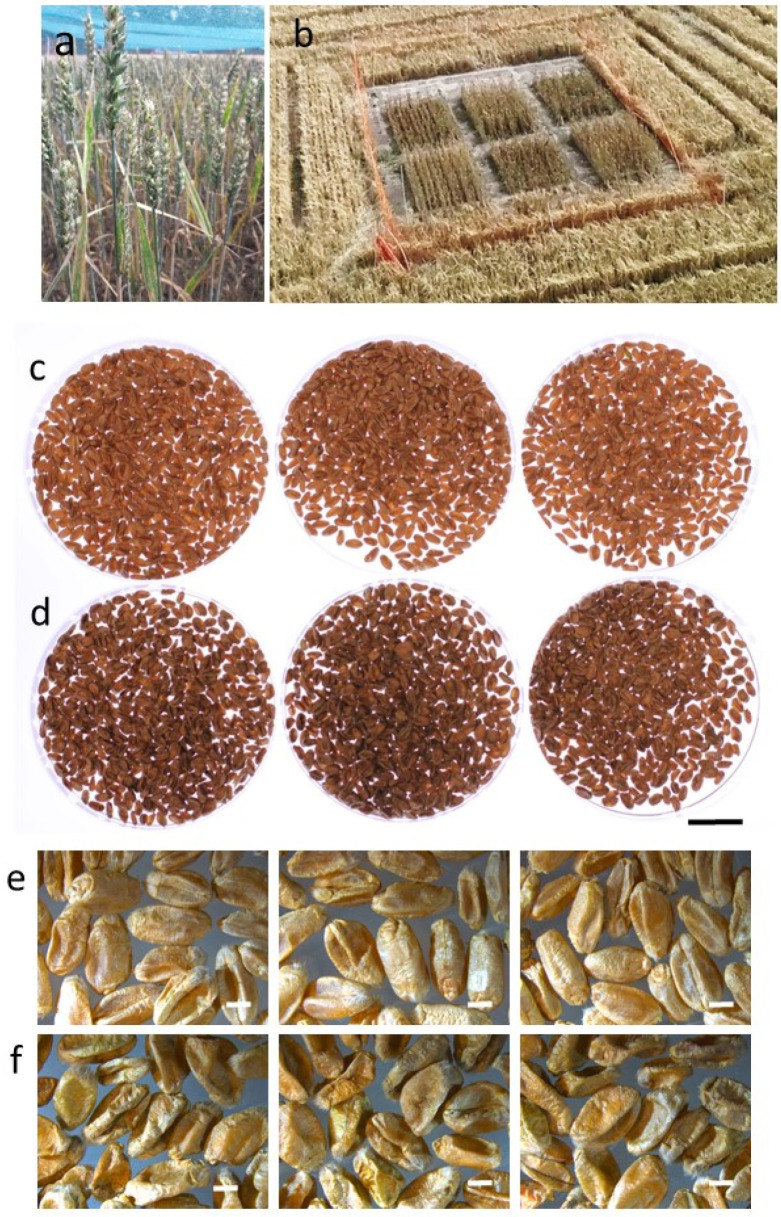
Photos on the field trial of *AsWRI1*-wheat and the harvested grain. (**a**) Developing spikes under insect proof net during flowering. (**b**) The field seen from above at grain maturity with the 2.5 m × 1.6 m plots with three replicates each of lines R3P8.12, null (control) and R3P8.10, homozygous with 12 gene inserts. (**c**) Triplicate batches of grains harvested from control and (**d**) R3P8.10 plants, black scale bar is 20 mm. (**e**) Close up photos of triplicate batches of grains from control and (**f**) R3P8.10, white scale bars are 2 mm.

Wheat lines with increased oil content in the grain were previously developed by heterologous endosperm-specific expression of oat (*Avena sativa*) endosperm *WRINKLED1* (*WRI1*) gene. These were named *AsWRI1*-wheat and characterized during seed development under greenhouse conditions [24]. To evaluate if the phenotypes that were observed under greenhouse conditions were also expressed under open field conditions, we performed a field trial in the south of Sweden (GPS coordinates (WGS84) 55.753535, 13.054206) during 2019. Grains from the *AsWRI1*-wheat line that showed the highest oil content in grains in previous experiments (line R3P8.10, homozygous with 12 gene inserts) and a control (line R3P8.12, azygous (null) segregant) were, therefore, multiplied in controlled daylight chambers during 2018. Grains were sown in the field in triplicate plots of 2.5 m × 1.6 m on 14 April 2019. Developing caryopses were harvested and flash frozen in liquid nitrogen after first removing the embryo. Intact grains were harvested at maturity on 28 August 2019. Plant height and general spike characteristics were measured just before the final grain harvest at maturity. An overview of the field trial and photos of the harvested grains are shown in Figure 1. Weather data (mean temperature, rainfall, solar radiation and relative humidity) during the growth period can be found in Supplementary Figure S1.

### 2.2. General Plant, Spike and Grain Characteristics from Field Grown and Greenhouse Grown Plants

**Figure 2:**
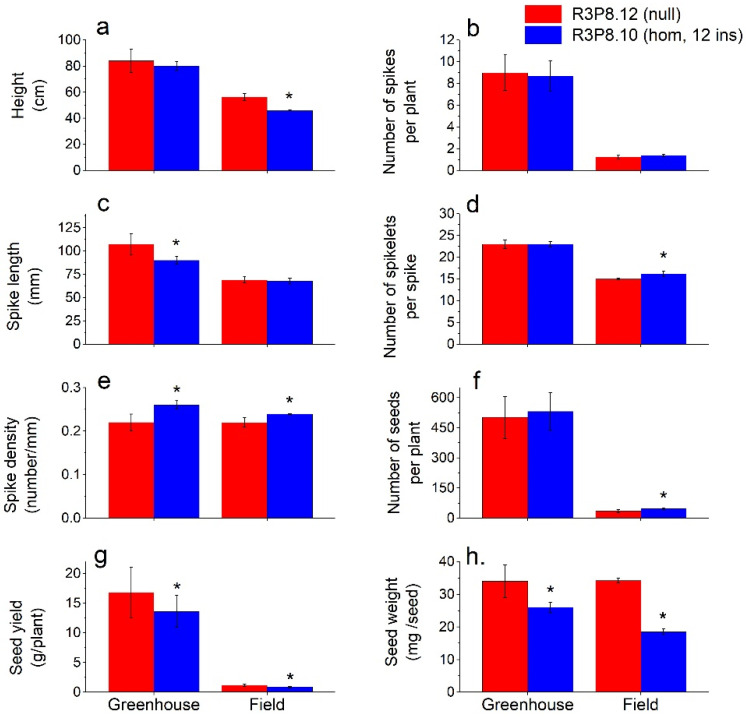
Comparison of plant and seed characteristics at grain maturity of greenhouse and field grown *AsWRI1*-wheat (line R3P8.12, null (control); line R3P8.10, homozygous with 12 gene inserts). Height of plants in cm including spike (**a**), number of spikes per plant (**b**), length of spikes in mm (**c**), number of spikelets per spike (**d**), spike density as number of spikelets per mm (**e**), number of seeds per plant (**f**), seed yield as gram per plant (**g**), and seed weight as mg per seed (**h**). The results shown are mean values from triplicate plots in field (based on the averages from 10 plants per plot) and 20 plants grown in a greenhouse ± standard deviation. Bars marked with asterisks are significantly different to control (null) according to Tukey’s test at *p* < 0.05.

To compare the general characteristics of the *AsWRI1*-wheat grown under greenhouse conditions with those under field conditions, we measured the following traits at grain maturity: plant height, number of spikes per plant, spike length, number of spikelets per spike, spike density, number of seeds per plant, yield per plant, and seed weight (Figure 2). The differences described below are significant at *p* < 0.05.

Under greenhouse conditions, we found no significant differences between the *AsWRI1*-wheat and control in the number of spikes per plant (8.9), the number of spikelets per spike (23), or the number of seeds per plant (500–530). The average plant height of *AsWRI1*-wheat was 4 cm shorter compared with the control, but only at a significance level of *p* < 0.07. A 16% reduction in spike length was observed in the *AsWRI1*-wheat as compared to the control (90 mm and 107 mm, respectively), which resulted in an increased spike density (number of spikelets/mm) in the *AsWRI1*-wheat giving a more compact spike appearance. Relatively large reductions in seed weight (−25%) and yield per plant (−20%) were found for the *AsWRI1*-wheat as compared to the control.

Under field conditions, we observed a clear reduction in plant height for the *AsWRI1*-wheat as compared to the control (46 cm and 56 cm, respectively). There was no difference in the number of spikes per plant in the field (1.3 on average). In contrast to greenhouse conditions, there was no difference between the *AsWRI1*-wheat and control in spike length (68 mm), but instead, a slightly higher number of spikelets per spike (16 and 15, respectively) which led to a small increase in spike density of the *AsWRI1*-wheat. Furthermore, the *AsWRI1*-wheat had a higher number of seeds per plant compared to the control (47 and 36, respectively) but a much-reduced seed weight (−46%), which led to a decreased yield on a per plant basis (−26%). Seed dimensions and other general characteristics from field grown *AsWRI1*-plants showed no difference in seed length, but slightly wider seeds, higher moisture content and much lower hardness index, as compared to the control (Supplementary Figure S2).

There were large differences in plant and seed characteristics between plants grown in the greenhouse and those grown in the field (Figure 2). The wheat plants grown under greenhouse conditions produced many more spikes per plant and longer spikes with more spikelets, as compared to the plants grown in the field. This, of course, gave a large difference in seed yield (resulting from the increased number and weight of seeds per plant), with much higher yields in the greenhouse grown material. However, whereas the weight of control seeds was identical between greenhouse and field grown plants, the reduction in seed weight of *AsWRI1*-wheat compared to the control was greater under field conditions than when grown in the greenhouse. Visual inspection showed that the mature *AsWRI1*-wheat grains were wrinkled with a shrunken endosperm compared to the control when grown under both greenhouse [24] and field conditions (Figure 1).

### 2.3. Lipid and Starch Contents of Endosperms from Developing Wheat Caryopses from Field Grown Plants

To monitor the accumulation of lipids and starch during seed development in field grown plants, caryopses were sampled at 11-, 19-, and 26-days post anthesis (dpa) and at seed maturity. To exclude the contribution of oil from the embryo, which is a small but oil dense tissue in all common cereals, the embryo was excised from caryopses. The remaining part, containing most of the starchy endosperm, aleurone, seed coat, and pericarp, was used for analyses (hereafter called the endosperm).

**Figure 3:**
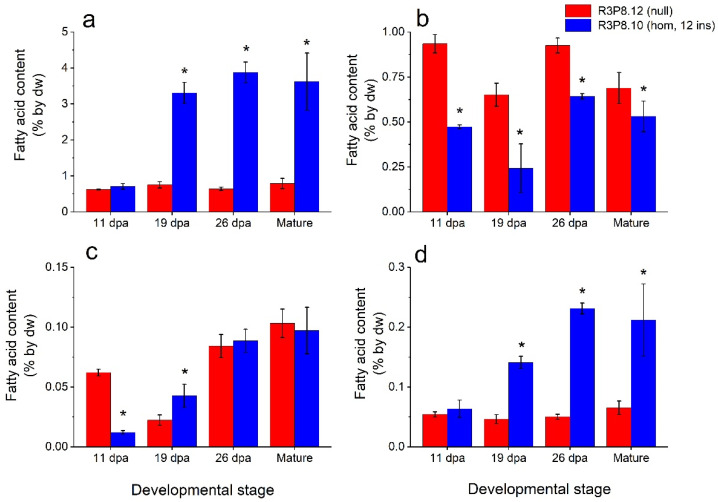
Lipid content based on amounts of the major fatty acids 16:0 (palmitic acid), 18:0 (stearic acid), 18:1 (oleic acid), 18:2 (linoleic acid), and 18:3 (linolenic acid) in the endosperms of developing caryopses from field grown *AsWRI1*-wheat (line R3P8.12, null (control); line R3P8.10, homozygous with 12 gene inserts). Levels of fatty acids are given as percentage of tissue dry weight (dw) of (**a**) triacylglycerol (TAG), (**b**) polar lipids (PL), (**c**) free fatty acids (FFA), and (**d**) other lipids at 11-, 19-, and 26-days post anthesis (dpa) and at seed maturity. Results are mean values of three replicates (i.e., plots) ± standard deviation. Bars marked with asterisks are significantly different to control (null) according to Fisher’s pairwise comparisons (*p* < 0.05).

The triacylglycerol (TAG) content of the endosperm of developing caryopses was increased four-fold in the *AsWRI1*-wheat as compared to the control from 19 dpa until maturity, but with no difference at 11 dpa (Figure 3a). The polar lipid content of the endosperms was lower, especially at the earlier developmental stages, in the *AsWRI1*-wheat, as compared to the control (Figure 3b). The free fatty acid content of the endosperms of *AsWRI1*-wheat was several-fold lower at 11 dpa, but almost two-fold higher at 19 dpa, after which no differences were observed compared to the control (Figure 3c). Finally, the content of other lipids in the endosperms was several-fold higher in the *AsWRI1*-wheat from 19 dpa onwards (Figure 3d). It should be noted that in *AsWRI1*-wheat the absolute levels of TAG were generally much higher than the other lipid fractions that were analyzed.

**Figure 4:**
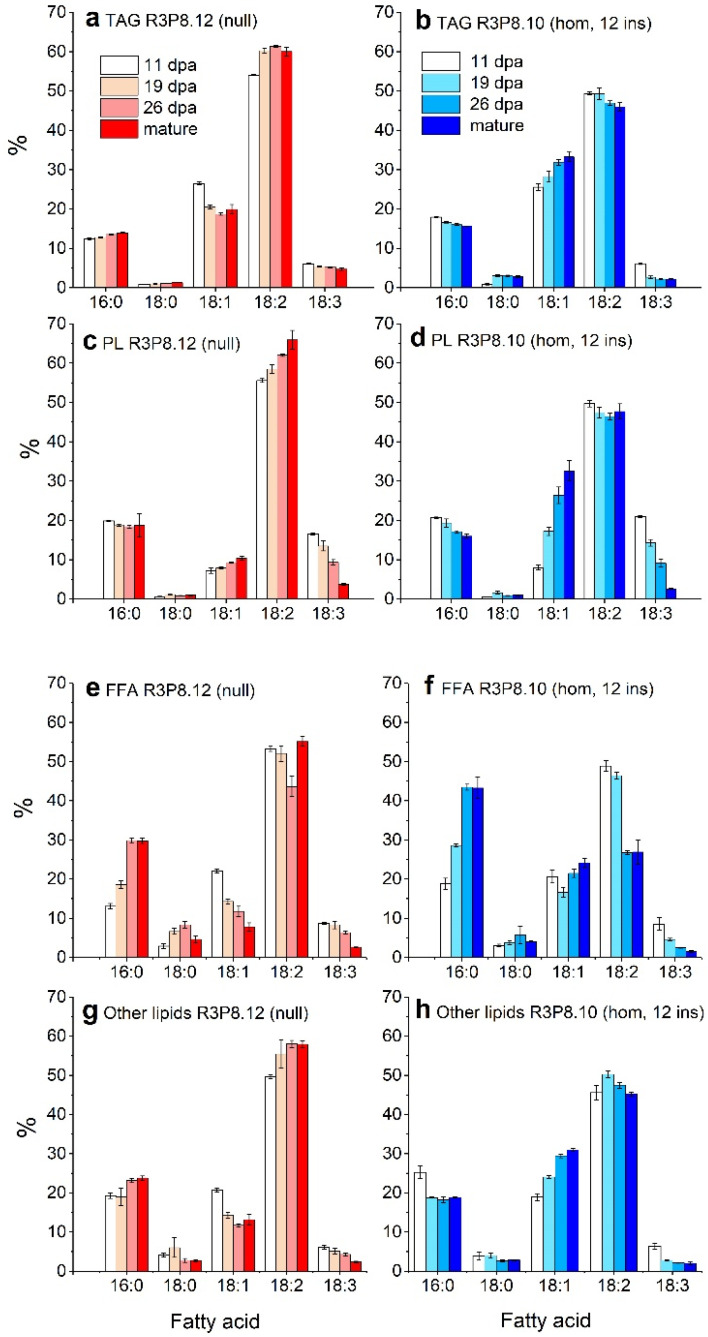
Fatty acid profiles of different lipid classes in the endosperm of developing caryopses from field grown *AsWRI1*-wheat (line R3P8.12, null (control) and line R3P8.10, homozygous with 12 gene inserts). (**a**,**b**) triacylglycerol (TAG), (**c**,**d**) polar lipids (PL), (**e**,**f**) free fatty acids (FFA), (**g**,**h**) other lipids. Profiles are given as percentage by weight of total major fatty acids (16:0 (palmitic acid), 18:0 (stearic acid), 18:1 (oleic acid), 18:2 (linoleic acid), and 18:3 (linolenic acid)) at 11-, 19-, and 26-days post anthesis (dpa) and at seed maturity. Results are mean values of three replicates (i.e., field plots) ± standard deviation.

The analyses of fatty acid profiles of the different lipid classes in developing endosperms (Figure 4) showed that the four major fatty acids in all lipids were palmitic acid (16:0), oleic acid (18:1), linoleic acid (18:2), and linolenic acid (18:3). In general, there were higher proportions of oleic acid and lower proportions of linoleic acid in all lipids in the *AsWRI1*-wheat, as compared to the control. Moreover, whereas the proportion of oleic acid in most lipids decreased during seed development in the control plants, it increased in the *AsWRI1*-wheat (for example, it increased from 8% to 33% in the polar lipids from 11 dpa to maturity). In the free fatty acid fraction, there was a large decrease in the proportion of linoleic acid in the *AsWRI1*-wheat between 19 dpa and 26 dpa (from 49% to 27%), which did not occur in the control.

**Figure 5:**
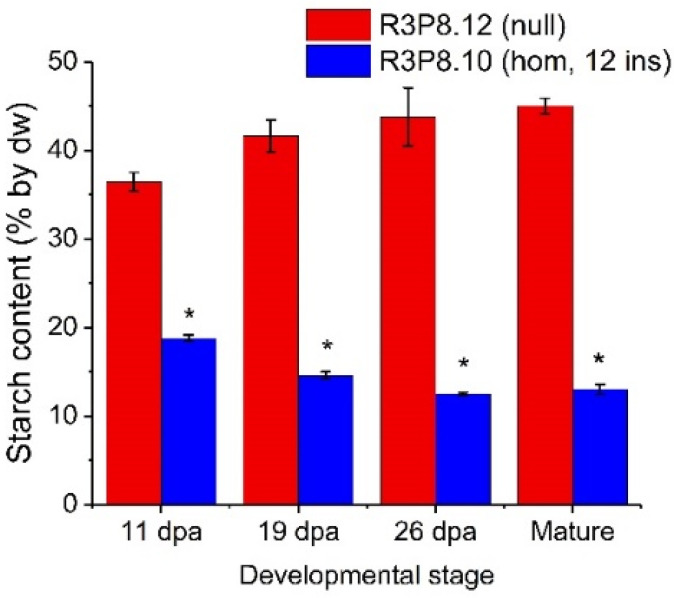
Starch content in endosperms of developing caryopses from field grown *AsWRI1*-wheat (line R3P8.12, null; line R3P8.10, homozygous with 12 gene inserts) at 11-, 19-, and 26-days post anthesis (dpa) and at seed maturity. Starch content is given as percentage of dry weight (dw). Results are mean values of three replicates (i.e., plots) ± standard deviation. Bars marked with asterisks are significantly different to control (null) according to Fisher’s pairwise comparisons (*p* < 0.05).

The starch content of the endosperm of developing caryopses was much lower in *AsWRI1*-wheat, as compared to the control (Figure 5). Even at 19 dpa the starch level in *AsWRI1*-wheat grains was approximately half, and by maturity, the level was below one third of the control.

### 2.4. Morphological Characterisation of Wheat Grains Using X-ray Microtomography

**Figure 6:**
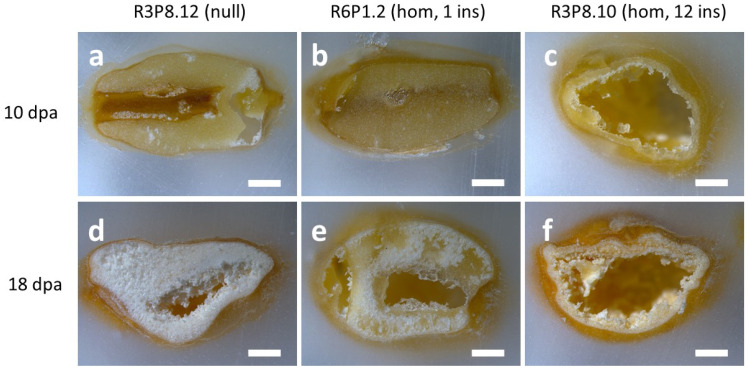
Photos of fixed and paraffin embedded caryopses from greenhouse grown *AsWRI1*-wheat. (**a**,**d**) line R3P8.12; null (control), (**b**,**e**) R6P1.2; homozygous with one gene insert, and (**c**,**f**) R3P8.10; homozygous with 12 gene inserts. (**a**–**c**) 10 days post anthesis (dpa) and (**d**–**f**) 18 dpa. Scale bars are 1 mm long.

We have previously reported that grains of *AsWRI1*-wheat are morphologically different from the control at a late developmental stage (26 dpa) and at maturity. To determine the morphological traits expressed in *AsWRI1*-wheat at earlier developmental stages, we first performed light microscopy analysis of caryopses sampled at 10 and 18 dpa from greenhouse grown plants of *AsWRI1*-wheat (three different lines; one with 12 gene inserts, one line with one insert, and the control). Comparison of sections of caryopses after fixation and embedding in paraffin showed that the morphology was very different in the *AsWRI1*-wheat, as compared to the control, as early as 10 dpa (Figure 6a–c). While the control at this stage had filled caryopses with a clear cellular structure throughout (Figure 6a), the cellular structure of the *AsWRI1*-wheat line with 12 gene inserts was only established along the periphery of the caryopses, leaving a central cavity (Figure 6c). However, the morphology of the grains of the *AsWRI1* expressing line with only one gene insert (Figure 6b) was similar to that of the control at this stage. At 18 dpa the interior of caryopses from control plants had a white and starchy appearance, which was present almost throughout the grain (Figure 6d). By contrast, the caryopses of both *AsWRI1* expressing lines had a greatly impaired seed filling (although this was less severe for the line with only one gene insert), with large cavities without cellular structure, while the white starchy endosperm was only present in the peripheral parts (Figure 6e,f). It can be noted that our previous work showed that these cavities were filled with a clear aqueous liquid during caryopses development which did not contain any lipids [24].

**Figure 7:**
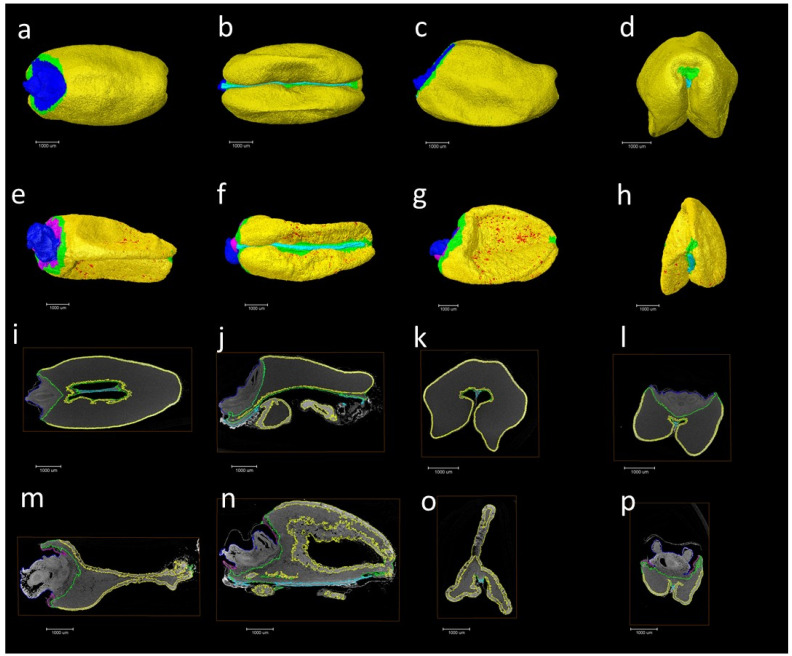
Pictures of seed surfaces (**a**–**h**) and examples of seed interiors (**i**–**p**) from X-ray microtomography analysis of mature wheat grains from field grown *AsWRI1*-wheat. Pictures show the surface from dorsal side (**a**,**e**), ventral side (**b**,**f**), lateral side (**c**,**g**), and front (**d**,**h**) of seeds. Pictures (**i**–**p**) show examples of interior parts of seeds from similar orientations. Grain from control plant (line R3P8.12, null) in (**a**–**d**,**i**–**l**), grain from *AsWRI1*-expressing line (R3P8.10, homozygous with 12 gene inserts) in (**e**–**h**,**m**–**p**). Color key: Yellow is aleurone, blue is germ and scutellum, green is endosperm, red is air pockets, cyan is the transfer bundle, and violet is the depleted region. Scale bars are 1000 μm.

**Figure 8:**
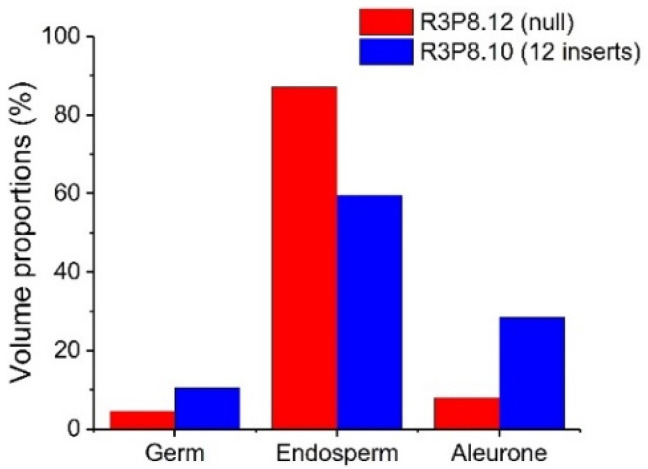
Proportions of volumes of different seed tissues determined by X-ray microtomography of mature wheat grains from field grown plants. *AsWRI1*-wheat line with 12 gene inserts (R3P8.10) and in control (null, R3P8.12). Results are from data from X-ray scans of single seeds.

The presence of elongated peripheral aleurone cells and multiple layers in the *AsWRI1*-wheat, as described previously [24], prompted us to investigate the seed morphology in more detail using X-ray microtomography on mature grains from field grown plants. This analysis gives three-dimensional images of the X-ray attenuation coefficient within the seed [26], which can be used to determine the volume of different seed tissues. The surfaces of grains from the images from 3D X-ray tomography clearly show that the *AsWRI1*-wheat grains are shrunken, wrinkled, and dotted with air pockets (Figure 7e–h) compared to the control (Figure 7a–d). Images of the seed interior from X-ray tomography also show that the structure of *AsWRI1*-wheat grains is more irregular (Figure 7m–p) compared to the control (Figure 7i–l). To further analyze the internal seed structures, quantification of the volumes of specific tissues was determined by summing the labeled areas in cross-sectional images extracted from the 3D images along the length of each grain. This analysis showed that the proportion of endosperm tissue out of the total seed volume was much lower in *AsWRI1*-wheat grains than in the control (60% and 87%, respectively), while the proportions of the germ (embryo and scutellum) and especially the aleurone tissue were greatly increased (Figure 8).

### 2.5. Lipid and Mineral Contents of Flour Fractions from Wheat Grains from Field Grown Plants

**Figure 9:**
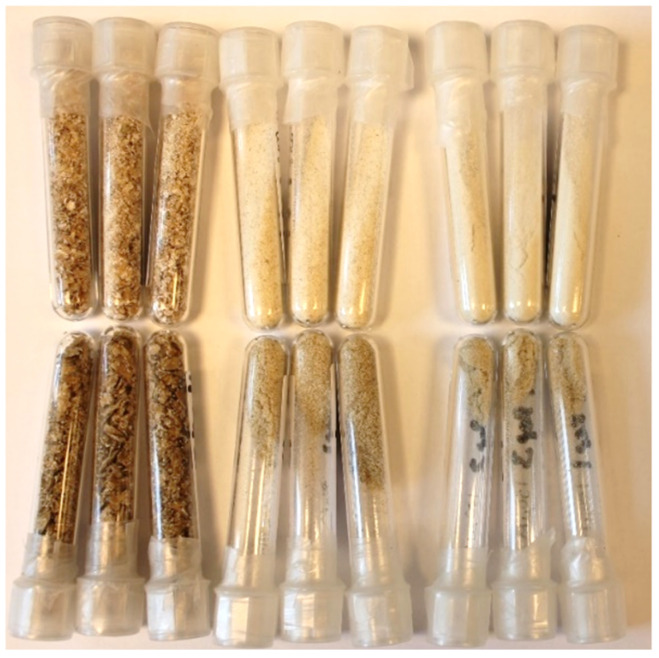
Photo of three different flour fractions from whole grains from field grown *AsWRI1*-wheat. The upper row shows flour from control (line R3P8.12, null), and the lower row shows flour from homozygous line with 12 inserts (line R3P8.10). The three tubes for each fraction represent the triplicate plots in field. Bran fraction (left), semolina flour (middle, 250 μm mesh), and endosperm flour (right, 150 μm mesh).

**Figure 10:**
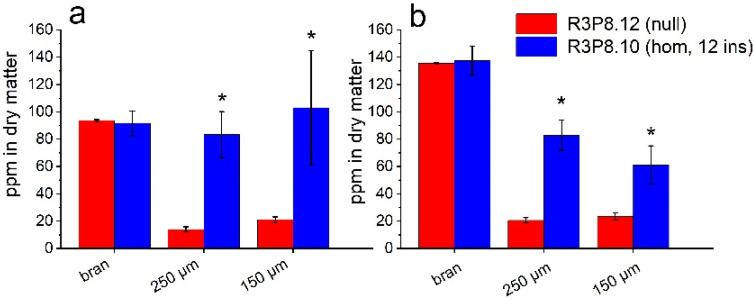
Mineral (Fe and Zn) content as ppm in dry matter of three different flour fractions (bran, 250 μm, and 150 μm mesh) of field grown *AsWRI1*-wheat at grain maturity (line R3P8.12, null (control); line R3P8.10, homozygous with 12 gene inserts). (**a**) Fe content. (**b**) Zn content. Results are mean values of three replicates (i.e., field plots) ± standard deviation. Bars marked with asterisks are significantly different to control (null) according to Fisher’s pairwise comparisons (*p* < 0.05).

To determine the distribution of components in the mature grain, samples of the *AsWRI1*-wheat and control line were milled using a small-scale roller mill to give three fractions: bran, semolina (coarse flour, 250 μm mesh) and fine flour (150 μm mesh). These flour fractions were used for the determination of lipids and minerals. Minerals are known to be concentrated in the aleurone layer and embryo of mature wheat grain and mineral (or ash) content is, therefore, used as a measure of the contamination of the white (starchy endosperm) flour with these tissues, which are usually recovered in the bran. The visual appearance of the flours from the *AsWRI1*-wheat was darker in all fractions compared to flours from control plants (Figure 9). The contents of iron (Fe) and zinc (Zn) were also several-fold higher in the semolina and endosperm flour fractions obtained from the *AsWRI1*-wheat, compared to the control, indicating contamination with aleurone and/or embryo tissues (Figure 10).

**Figure 11:**
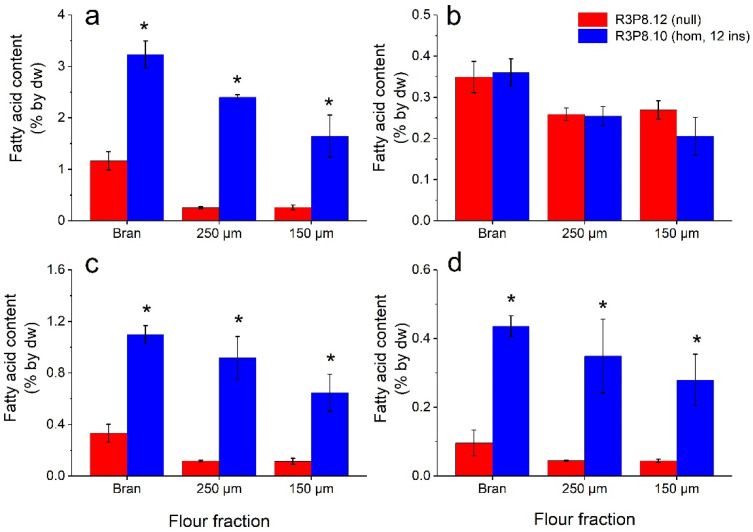
Lipid content (based on amounts of major fatty acids 16:0 (palmitic acid), 18:0 (stearic acid), 18:1 (oleic acid), 18:2 (linoleic acid), 18:3 (linolenic acid) and 20:1 (eicosenoic acid)) in three different flour fractions (bran, 250 μm, and 150 μm mesh) from mature wheat grains from field grown *AsWRI1*-wheat (line R3P8.12, null (control); line R3P8.10, homozygous with 12 gene inserts). Levels of fatty acids are given as a percentage of tissue dry weight (dw) from (**a**) triacylglycerol (TAG), (**b**) polar lipids (PL), (**c**) free fatty acids (FFA), and (**d**) other lipids. Results are mean values of three replicates (i.e., plots) ± standard deviation. Bars marked with asterisks are significantly different to control (null) according to Fisher’s pairwise comparisons (*p* < 0.05).

The lipid analyses clearly showed that all three flour fractions from the *AsWRI1*-wheat grains had several-fold higher contents of TAG, free fatty acids, and other lipids as compared to in control (Figure 11). The highest levels were found in the bran fraction, and the lowest in the fine (150 μm) flour fraction (Figure 11a,c,d). However, there was no difference in contents of polar lipids in the flours from *AsWRI1*-wheat compared to the control (Figure 11b). It should be noted that the absolute levels of free fatty acids in *AsWRI1*-wheat were much higher in the flour fractions as compared to the intact endosperms, with levels of above 1% and below 0.1% by dw, respectively (Figure 3c and Figure 11c). Visual inspection of the thin layer chromatography plates used for the separation of different lipid classes showed that the increase in ‘other lipids’ observed in *AsWRI1*-wheat, which was also seen in the intact endosperms of developing caryopses, was mainly due to a lipid class migrating to a position between TAG and free fatty acids (Supplementary Figure S3).

Similarly, as seen in the fatty acid profiles of lipids in the intact endosperm of developing caryopses (Figure 4), the fatty acid profiles of the lipids in the flour fractions obtained from the *AsWRI1*-wheat all had a higher proportion of oleic acid and a lower proportion of linoleic acid (Supplementary Figure S4). No large differences in the fatty acid profiles of lipids were found in the different flour fractions in any of the wheat lines. Interestingly, the fatty acid profiles of the lipids that were analyzed in the flour fractions were very similar to those present in the lipids of the intact endosperms of mature grains, except for the free fatty acids in the *AsWRI1*-wheat (in which the proportion of palmitic acid was about half and the proportion of linoleic acid much higher in the flour).

## 3. Discussion

In our previous work, we explored how genetic modification of wheat to express a regulatory gene in central carbon metabolism from a high-oil oat variety in the developing starchy endosperm affected grain structure and composition [24]. We showed that the endosperm-specific expression of a WRINKLED1 gene, which is expressed in developing oat endosperm [21,23], resulted in a several-fold increase in the content of oil and sugar while the starch content was greatly reduced compared to the control [24]. In this study, we investigated how this transgenic wheat with induced oil accumulation performed under open field conditions in the south of Sweden. We also determined the effects of the transgenic expression on the structure of the developing and mature grains by light microscopy and high-resolution X-ray microtomography. Finally, we determined lipid and mineral compositions of flour and bran fractions obtained by roller milling of the mature grains.

We initially compared the general characteristics of plants and mature seeds grown under greenhouse and field conditions. This showed that some of the differences observed between the *AsWRI1*-wheat and control wheat were more pronounced when grown under field conditions than under greenhouse conditions. For example, while the height of the *AsWRI1*-wheat plants tended to be lower than that of the control plants, this was more clearly observed under field conditions where up to 10 cm difference occurred. Similarly, the reduction in grain weight of *AsWRI1*-wheat was greater under field than glasshouse conditions. This is explained by the fact that the plants grown in fertilized soil in pots in the greenhouse were more vigorous, with more spikes per plant and thus higher yields. It is, therefore, important to determine the performance of modified types of wheat under field conditions to evaluate their potential for exploitation by breeders.

Lipid analyses of developing and mature caryopses showed that the increased oil content and changes in oil composition observed in *AsWRI1*-wheat under greenhouse conditions [24] were retained under field conditions. The TAG content in endosperms of mature grains of *AsWRI1*-wheat was 3.5% by dw compared with below 1.0% by dw in the control, and the increased TAG content was associated with an increased proportion of oleic acid (18:1). An increased proportion of oleic acid in TAG has previously been shown to be a signature of increased capacity of oil synthesis in plants, for example in oat grains [10], but also when inducing oil synthesis in leaf tissue through transient gene expression of *AsWRI1* [22]. The starch content was much reduced in endosperms of developing and mature caryopses, accounting for less than 15% by dw in mature grains of *AsWRI1*-wheat, compared with 45% by dw in the control. The wrinkled seed phenotype observed under greenhouse conditions was also observed in the field grown material and is probably caused by the observed reduction in starch content of grains. This seed phenotype is similar to the classical wrinkled pea, which has a mutation in starch synthesis [27]. Our results from lipid analyses showed that the strategy of inducing an increased oil content in wheat grains by endosperm-specific expression of *WRI1*, which is a gene coding for a transcription factor that reallocates carbon into storage lipids in the form of TAG, is functional also under field conditions.

Seeds comprise several tissues that differ in their compositions, including in terms of their concentrations of components that affect the nutrition and health of consumers. The proportion of the tissues in the mature grain, therefore, affects seed quality and end use [28]. Similarly, variation in the proportions and composition of tissues within the seeds of a single crop species, such as wheat grains, also has impacts on grain quality and end use. In wheat grains, as in most other cereals, the TAG accumulates mainly in the embryo and aleurone, which together form only a small proportion of the mature grain (about 10% by dw [5]) with the major starchy endosperm tissue (80–85% by dw) having a very low TAG content [6]. However, oats differ from all other cereal crops by having a wide range of variation in the content of storage lipids (TAG) in the starchy endosperm tissue [10]. This results in high oil contents in some varieties, up to 18 % by grain weight [12]. There are also gradients in the content of oil in the starchy endosperm, being higher in the subaleurone cells [9]. These cells also differ from the central starchy endosperm cells in other respects, being rich in protein (up to 40% dw) and low in starch [2,29,30]. The difference in composition between the subaleurone and central starchy endosperm cells may result from the fact that the former have a different origin, being derived from anticlinal divisions of the aleurone cells which continue to divide up to about 14 days after anthesis [31].

The aleurone layer of cereal grains is the outermost layer of the endosperm, comprising a single layer of cells in wheat. It is rich in protein, lipids, minerals, B vitamins and phytochemicals and, hence, is a potential target for breeding for the increased nutritional quality of wheat [25,32]. However, it should be noted that the aleurone forms part of the bran fraction when the grain is milled and is not present in white flour [33]. The aleurone layer of the *AsWRI1*-wheat differs from that in control plants, comprising elongated aleurone cells which were sometimes present in multiple layers [24], as compared to the single layer of cuboid shaped cells in the control, as is usual for wheat [25,34]. In the present study, we used high-resolution X-ray microtomography of mature individual grains to determine the volume proportions of the different seed tissues. Our new data complement the previous studies and show that the proportion of the aleurone was much increased, while that of the starchy endosperm instead was much decreased, in the *AsWRI1*-wheat grains as compared to control (28% and 8% aleurone, and 60% and 87% starchy endosperm, respectively). Our results also demonstrate that X-ray based characterization methods can be effective, non-destructive tools to provide additional insights into seed morphology that are not possible using conventional microscopy. X-ray microtomography has been used in previous work to determine the proportions of different tissues of quinoa seeds and maize kernels [35,36] and also the microstructure of wheat-based food [37,38]. In the current work, 3D images of the external and internal structures of the grains were obtained by X-ray tomography allowing the volumes of the different grain tissues to be quantified.

The reduced grain weight in *AsWRI1*-wheat, which results from decreased starch content and volume of the starchy endosperm, is clearly not appropriate for exploitation by breeders. In a recent study, a ‘push-pull-protect’ approach was used in wheat, by combining endosperm-specific expression of *WRI1* with genes coding for an acyltransferase involved in TAG assembly and an oleosin important for proper packaging of oil. This resulted in a significantly increased oil content in the grains while the starch content and seed weight were retained [39,40]. A comparison between these previous results with ours allows for an increased understanding of how the metabolism of the wheat endosperm is affected by different biotechnological approaches to develop wheat grains with increased oil content. Our previous analyses of *AsWRI1*-wheat endosperms showed, in addition to increased levels of TAG and transcripts encoding functions in fatty acid synthesis and oleosins, increased levels of free fatty acids throughout grain development and transcripts coding for genes involved in the degradation of lipids and fatty acids [24]. This is in contrast to in the ‘push-pull-protect oil-wheat’ in which none of the genes encoding functions in lipid degradation were up-regulated [40]. Altogether, this indicates that the expression of the transcription factor WRI1 alone causes a carbon metabolism imbalance in the wheat endosperm such that the starch synthesis is reduced, but the TAG synthesis machinery is not able to utilize all available fatty acids from de novo synthesis. This can result in the accumulation of free fatty acids that can be cytotoxic in plants and algae due to their high hydrophobicity [41,42,43]. Furthermore, although transcripts encoding oleosins were upregulated in *AsWRI1*-wheat [24] the increase may not be sufficient for the proper packaging of TAG. This could result in the degradation of both TAG and excessive fatty acids. Transcripts encoding enzymes involved in the turnover of TAG and fatty acids have previously also been observed with ectopic WRI1 expression in leaves of both monocot and dicot plant species [22,44,45].

The differences in the spatial distribution of components between and within the different tissues of the cereal grain have implications for the end uses, such as milling [2]. To determine the impact of expression of *AsWRI1* on grain milling, we prepared three fractions from the mature grains of field grown plants using a laboratory roller mill (which has a similar action to commercial mills). These were bran, semolina (coarse flour, 250 μm mesh) and fine flour (150 μm mesh). We also determined the contents of minerals in these fractions, as minerals are concentrated in the aleurone layer and embryo, so mineral content (measured as ash) is routinely used to measure the purity of white flour fractions during milling. Analyses of the two major mineral micronutrients, Fe and Zn, showed high levels in the two flour fractions from *AsWRI1*-wheat, but not in those of the control wheat. This ‘bran contamination’ probably resulted from the wrinkled phenotype and the higher proportion of aleurone cells in the *AsWRI1*-wheat. Further, our analyses of lipid content showed that all flour fractions had increased TAG content in *AsWRI1*-wheat compared to in the control, with the bran fraction having the highest level. The aleurone layer and embryo of wheat are both rich in TAG (approximately 6% and 20% by dw, respectively) compared to the starchy endosperm cells with levels below 0.5% by dw [6,46]. Hence, the bran contamination of the flour fractions of the *AsWRI1*-wheat would also result in the presence of TAGs derived from the aleurone layer and embryo, so the true contribution of lipids from the starchy endosperm cannot be determined.

The TAG levels in the flour fractions from *AsWRI1*-grains were comparable but slightly lower than those found in the intact mature grain endosperms. By contrast, the levels of FFAs were much higher in the flour fractions from *AsWRI1*-grains compared to in intact developing endosperms, which indicates that some oxidation of TAG and other lipids may have occurred during milling and/or storage of the flour. Lipids in intact non-germinated cereal grains are usually not oxidized, but this process can be activated by endogenous lipases (i.e., present in the germ and aleurone) during processing, such as milling. This is undesirable because the degradation products give a bitter taste and a reduced shelf life. However, it is not usually a problem with white wheat flour, which can be stored for up to a year before being used for baking. By contrast, wholemeal flour (which has a higher lipid content) is usually stored for a shorter period, while lipid-rich cereal grains, such as oats and oil-rich germ from wheat milling, need to be heat-treated to inactivate lipases and lipoxygenases prior to storage [47,48]. Recent work also showed that down-regulation of lipoxygenase activity through transgenic modification improved the grain storability of wheat [49].

## 4. Materials and Methods

### 4.1. Plant Material and Growth Conditions

Wheat expressing the *AsWRI1* under the control of the starchy endosperm-specific HMW1Dx5 promoter and the HMW and 35S terminator was developed as previously described [24]. Seeds from T3 generation of lines R3P8.10 (homozygous with 12 gene inserts) and R3P8.12 (a null) were multiplied in controlled daylight chambers during 2018 (Biotronen SLU, Alnarp, Sweden) by cultivation in 3.5 L pots with soil fertilized with 3M Plus Basacote (Compo Expert, Muenster, Germany). The temperatures were 21 °C (day) and 18 °C (night) with a 16 h photoperiod provided by natural light supplemented with Son-T PIA 400 W sodium lamps (Philips, Amsterdam, Netherlands) when the natural light fell below 200 μmol m^−2^ s^−1^ photosynthetically active radiation, at a relative humidity of 70%. Grains were harvested from plants at maturity and threshed by hand.

Seeds were sown in a field in Borgeby, Sweden (GPS coordinates (WGS84) 55.753535, 13.054206) in triplicate plots of 2.5 m × 1.6 m on 14 April 2019. The seed densities were adjusted to compensate for the lower germination rate of the *AsWRI1*-wheat as compared to control (460 seeds/m^2^ and 350 seeds/m^2^, respectively). Spring barley was sown as a 10 m wide pollen barrier around the genetically modified wheat. To further minimize the risk of spreading the genetically modified wheat to the surroundings by animals or insects, the field was covered in fiber cloth until plants had germinated and established and covered with an insect proof net during the flowering period. The field was fertilized with NPK 26-3-4 (400 kg/ha) and calcium nitrate (320 kg/ha). Weeds were treated using the herbicides Event Super (Bayer Crop Science, Leverkusen, Germany) and Express (DuPont, Wilmington, DE, USA) at concentrations of 1 L and 15 g per ha, respectively, through leaf application. The field was irrigated twice with 25 mm during the cultivation period, on 10 and 17 July. Spikes were tagged at anthesis and caryopses were manually harvested at different developmental stages and snap frozen in liquid nitrogen, after first having removed the embryo from each grain using a scalpel. Grains from single plots were pooled and frozen seed tissues were then stored at −80 °C. Ten randomly selected plants from each plot were harvested at maturity (21 August 2019) for the determination of plant height (including spike length) and spikes were then cut from the straw and stored in paper bags for later determination of spike and grain characteristics. The final harvest of grains from each plot was on 28 August 2019. Weather data during the growth period was downloaded from https://www.slu.se/fakulteter/nj/om-fakulteten/centrumbildningar-och-storre-forskningsplattformar/faltforsk/vader/lantmetv/ (18 March 2022), based on the weather station Borgeby (Nr 20947) and can be found in Supplementary Figure S1.

Plants to be used for fixation and microscopy analyses of developing caryopses were grown in 3.5 L pots in a greenhouse at 21 °C (day) and 18 °C (night) with a 16 h photoperiod provided by natural light supplemented with sodium lamps. Plants were irrigated manually three times a week. Three lines were used for this analysis: line R3P8.12, null (control), R6P1.2 (homozygous with one gene insert), and R3P8.10 (homozygous with 12 gene inserts).

### 4.2. Grain Characteristics Analyses and Flour Fractionation

Grain weights were determined by weighing and counting all grains from ten individual plants from each plot in the field trial using a seed counter (Contador, Pfeuffer, Kitzingen, Germany). Harvested grains from each plot were then pooled and used for further analyses. The lengths and widths of mature seeds were measured using the MARVIN-Digital Seed Analyser (MARViTECH GmbH, Wittenburg, Germany) and software Marvin 4.0. Grain hardness and moisture content were determined using the Perten Single Kernel Characterisation System (SKCS) 4100 (Calibre Control International Ltd., Warrington, UK) following the manufacturer’s procedure. Three hundred grains from each plot were used for each analysis (Perten Instruments, Calibre Control International Ltd., UK).

Ten grams of seed from each transgenic and control line were analyzed using a NIRFlex solids BÜCHI machine (BÜCHI Labortechnik AG, Flawil, Switzerland) and the NIRWare 1.2 Software, using an internal calibration for seed moisture content. The moisture content was adjusted to 15.5% by the addition of water and samples were conditioned for 2 h at room temperature on a Rolling Shaker before being milled using a Micro Scale Labmill FQC-2000 (METEFÉM Szövetkezet, Budapest, Hungary). This small-scale roller mill has a similar action to large commercial roller mills. Wholegrain flour from the mill was sieved into three fractions corresponding to bran, a semolina coarse flour of ≤250 μM and white fine starchy flour of ≤50 μM. The flours were stored at room temperature for several weeks prior to analyses.

### 4.3. Starch, Lipid and Mineral Analyses

Grain tissues from the field were stored at −80 °C and ground into a fine flour using steel containers and beads (Retsch, Haan, Germany) chilled in liquid nitrogen at 30 Hz (15 s, 12 mm steel bead) to prevent thawing. The flour was freeze-dried and stored at −20 °C until further analysis.

Starch was determined enzymatically on 35–55 mg freeze-dried flour using the Total Starch Determination Kit K-TSTA (Megazyme, Wicklow, Ireland) according to the standard procedure, but with sugars first removed from the flour with 80% aqueous EtOH according to instructions.

Total lipids were extracted from 60-90 mg freeze-dried flour with chloroform according to a modified method of Bligh and Dyer [50] using 0.15 M acetic acid instead of water. Lipids in aliquots corresponding to 25 mg dw were separated using thin layer chromatography (TLC) plates (silica pore diameter 60 Å, Merck, Darmstadt, Germany) in a medium containing heptane:diethyleter:acetic acid in volume ratios 70:30:1. Lipids were visualized under UV-light using 0.05% primulin spray as lipophilic dye [51], identified by Rf values and authentic standards and scraped off the plates into glass tubes. The silica was dried on warm sand under nitrogen gas and the lipids were then methylated to fatty acid methyl esters (FAMEs) in 2 mL of 2% H_2_SO_4_ at 90 °C fzzor 45 min with heptadecenoic acid methyl ester (17:0-ME) being added as internal standard. FAMEs were extracted by addition of 1 mL heptane and 2 mL H_2_O followed by vortexing and centrifugation at 3000 rpm for 2 min. FAMEs in the heptane phase was finally separated on an Intuvo DB-23 column (30 m, 0.25 mm inner diameter, 0.25 μm film thickness) using a gas chromatograph split between a mass spectrometer for the identification of FAMEs and a flame ionization detector for quantification of FAMEs (Intuvo GC and MSD5977, Agilent Technologies, Santa Clara, CA, USA). The contents of different lipids were calculated as the total weight of all fatty acid methyl esters in each lipid. Flour fractions were stored at room temperature for several weeks after milling and prior to lipid analyses. Flour fractions were then freeze-dried and lipid analysis was performed as described above.

The mineral content in flour fractions was determined using Inductively Coupled Plasma-Optical Emission Spectroscopy (ICP-OES) 5900 (Agilent Technologies, Santa Clara, CA, USA). Samples were oven-dried at 80 °C overnight, weighed and digested using a mixture of nitric acid and perchloric acid (85:15 *v*/*v*) in open tube digestion blocks, followed by programmed heating digestion: 60 °C for 180 min, 100 °C for 60 min, 120 °C for 60 min, 175 °C for 90 min and 50 °C until dry. The acids were removed by volatilization and the residue dissolved in nitric acid (5% *v*/*v*). The elements were detected with Optima 7300 DV ICP-OES. The analysis was strictly monitored using certified external standards alongside in-house standard materials. Standards and check samples are monitored and recorded using Shewhart Control Graphs and computer-based quality control packages.

### 4.4. Fixation of Caryopses and Light Microscopy

Developing caryopses from *AsWRI1*-wheat lines (R3P8.12, null; R6P1.2, homozygous with one gene insert; R3P8.10, homozygous with 12 gene inserts) were harvested at 10 and 18 days from plants grown in the greenhouse. The intact caryopses were placed in glass tubes with a freshly prepared fixation solution of 4% (*w*/*v*) paraformaldehyde in 0.1 M Sörensens’s phosphate buffer pH 7.2 overnight in 8°C on a shaking table. Fixed caryopses were then rinsed 3 × 15 min in 0.1 M Sörensens’s phosphate buffer pH 7.2 and stored at 4 °C until the next step. Fixed caryopses were dehydrated by washing in a series of ethanol (from 30% to 100%) followed by clearing in a series of ethanol:xylene (2:1, 1:1, then 100% xylene). Samples were finally infiltrated in paraffin (Histowax Special, Histolab Products AB, Göteborg, Sweden) by incubating at 55 °C (3 × 24 h) before being cast in molds. Longitudinal sections were cut from the paraffin embedded caryopses using a microtome and pictures of blocks were then taken with a microscope (Leica M165FC with camera DFC450 C, Leica, Wetzlar, Germany).

### 4.5. X-ray Microtomography

Individual grains were wrapped in parafilm and placed in a thin-walled plastic tube that was secured on the sample holder of a Zeiss XRM520 (Carl Zeiss X-ray Microscopy, Inc., Pleasanton, CA, USA) at the 4D Imaging Lab at Lund University. For each grain, 1601 X-ray projections were obtained over 360° using a 50 kV source voltage. The projections were reconstructed, using the Zeiss reconstructor software (version 12), into three-dimensional image volumes, in the form of 16-bit image stacks, with cubic voxels of 4.2 μm side length for the control grains and 4.8 μm for the *AsWRI1*-wheat grain. The different voxel dimensions relate to the different sizes of the grains.

### 4.6. Analysis of Volumetric Images

Reconstructed X-ray tomography image stacks were spatially cropped and downsampled to 8-bits using Drishti Paint [52]. The 8-bit images were imported into Amira (2019, Thermo Fischer Scientific, Berlin, Germany) and smoothed using a 1 voxel radius 3D median filter. Each anatomical region of each grain was manually labeled using the ‘brush’ tool in the Amira Segmentation Editor on approximately 30 individual slices along the length of each grain. Biomedisa [53] was then used to interpolate the manual labels to the remainder of the volume. If the interpolation results did not accurately indicate the regions at some points along the grain length, additional slices were manually labeled at those points before re-running the interpolation to produce a predominantly faithful segmentation. However, air pockets and the depleted region in the *AsWRI1*-wheat grain could not be satisfactorily labeled using interpolation and required manual labeling by the ‘brush’ and ‘magic wand’ tools on a slice-by-slice basis. The volumes of each labeled region were obtained from the Material Statistics report, while Surface View and Slice modules were used to produce visualizations of the segmentation results.

### 4.7. Statistical Analyses of Data

Data for metabolites and plant and grain characteristics were analyzed using one-way analysis of variance (ANOVA) followed by Tukey’s or Fisher’s tests (*p* < 0.05, LSD) to compare the means of treatment groups (Minitab 18, Minitab, State College, PA, USA). Fisher’s test was used when predefined pairwise comparisons within data sets were of interest (for example between transgenic line and control, for each developmental stage).

## 5. Conclusions

The use of transgenic technologies to increase our understanding of gene functions can play an important role in crop improvement, as a complement to conventional breeding methods. Genes encoding transcription factors have been identified as controlling domestication traits, but also as controlling varietal differences in several crops including wheat [54] and are, therefore, interesting targets for plant breeding. Targeting transcription factors that are known to regulate central carbon metabolism could be of interest for widening the range of variation in composition and end use quality of wheat. In this work, we studied the effect of overexpression of the transcription factor *AsWRI1* in the wheat endosperm under field conditions. Our results showed that (i) the increased triacylglycerol content and changed morphology observed in *AsWRI1*-wheat grains as compared to control plants under greenhouse conditions was also exhibited under field conditions and (ii) flours produced from *AsWRI1*-wheat grains had increased oil content but were contaminated with the bran due to the effects of the changed morphology on milling. The reduced grain weight of *AsWRI1*-wheat observed under both greenhouse and field conditions shows that transcription factors may drastically change gene expression patterns leading to widespread effects on metabolism and grain composition. From a practical perspective, the development of efficient biotechnological strategies to overcome possible undesired effects, such as reduced content of major storage compounds and reduced seed weight may, therefore, be required to produce novel types of wheat that combine improvements in composition and end use quality with good agronomic performance and high yields.

## Data Availability

Not applicable.

## Supplementary Materials

The following supporting information can be downloaded at https://www.mdpi.com/article/10.3390/plants11070889/s1, Figure S1: Weather data during the growth period (137 days in total, from 14 April to 28 August 2019) in field (Borgeby, Sweden, GPS coordinates (WGS84) 55.753535, 13.054206) during 2019. (a) Daily mean temperature at 1.5 m above ground, (b) rainfall, (c) solar radiation, (d) relative humidity. Figure S2: Length, width, moisture content, and hardness index of mature grains from field grown AsWRI1-wheat; Figure S3: Photo of thin layer chromatography plates with separated lipid classes from flour fractions of field grown *AsWRI1*-wheat; Figure S4: Fatty acid profiles of lipid classes in three different flour fractions (bran, 250 μm, and 150 μm mesh) from mature wheat grains from field grown *AsWRI1*-wheat.
