# Supplementary material for: Characterisation of Grains and Flour Fractions from Field Grown Transgenic Oil-Accumulating Wheat Expressing Oat WRI1
Source: Plants (Basel). 2022 Mar 26;11(7):889. doi: 10.3390/plants11070889 (PMC9002947; doi:10.3390/plants11070889)
Supplement: Supplementary file 1 [file plants-11-00889-s001.zip › plants-1644654-supplementary.pdf]

plants

MDPI

Supplementary material

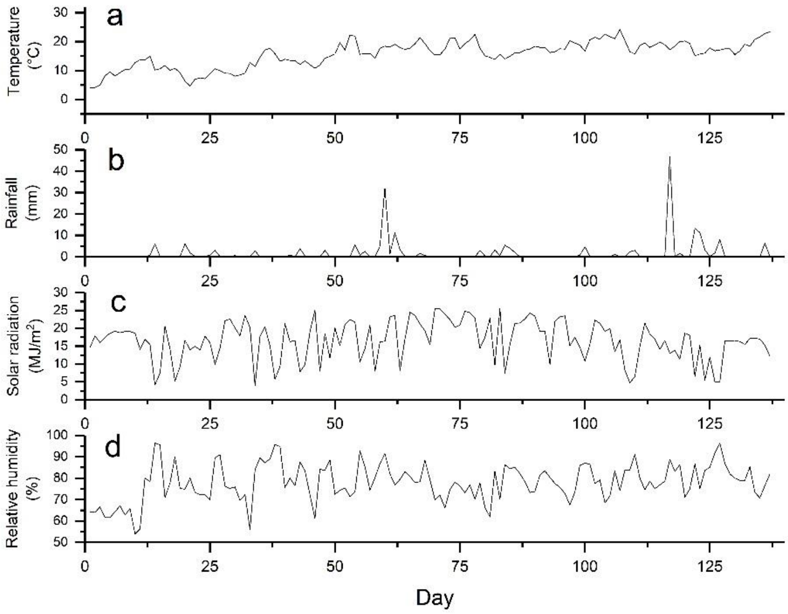

Figure S1: Weather data during the growth period (137 days in total, from 14 April to 28 August, 2019) in field (Borgeby, Sweden, GPS coordinates (WGS84) 55.753535, 13.054206) during 2019. (a) Daily mean temperature at 1.5 m above ground, (b) rainfall, (c) solar radiation, (d) relative humidity.

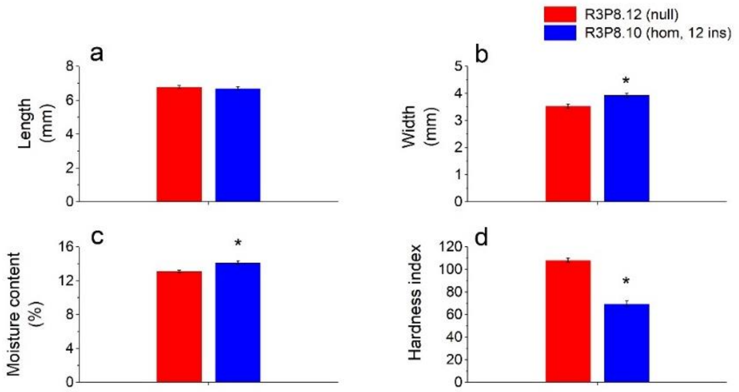

Figure S2. Grain characters of field grown AsWRI1-wheat (line R3P8.12, null (control); line R3P8.10, homozygous with 12 gene inserts). (a) Length, (b) width, (c) moisture content, and (d) hardness index. Results shown are mean values from triplicate plots in field (based on the averages from technical triplicates from each plot á approximately 100 seeds) ± standard deviation. Bars marked with asterisks are significantly different to control (null) according to Tukey´s test at $p < 0.05$.

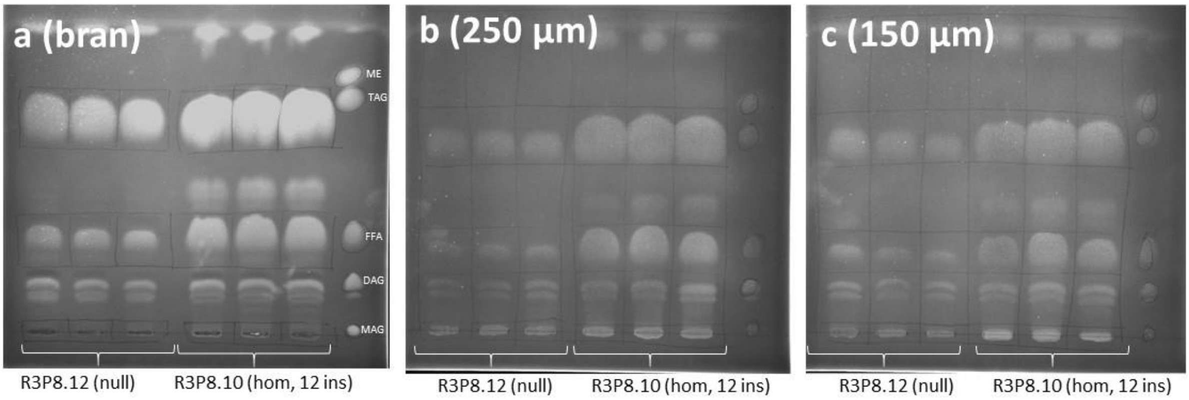

Figure S3. Photo of thin layer chromatography plates with separated lipid classes from flour fractions of field grown AsWRI1-wheat. The three different flour fractions are (a) bran, (b) 250 µm, and (c) 150 µm. Lipids were visualized with primuline stain under UV-light. Pencil lines are drawn around different lipid spots. The lane to the right in all plates show the lipid references with monoacylglycerol (MAG), diacylglycerol (DAG), free fatty acids (FFA), triacyglycerol (TAG), and methyl esters (ME). Results shown are three replicates (i.e. field plots) from line R3P8.12, null and line R3P8.10, homozygous with 12 gene inserts.

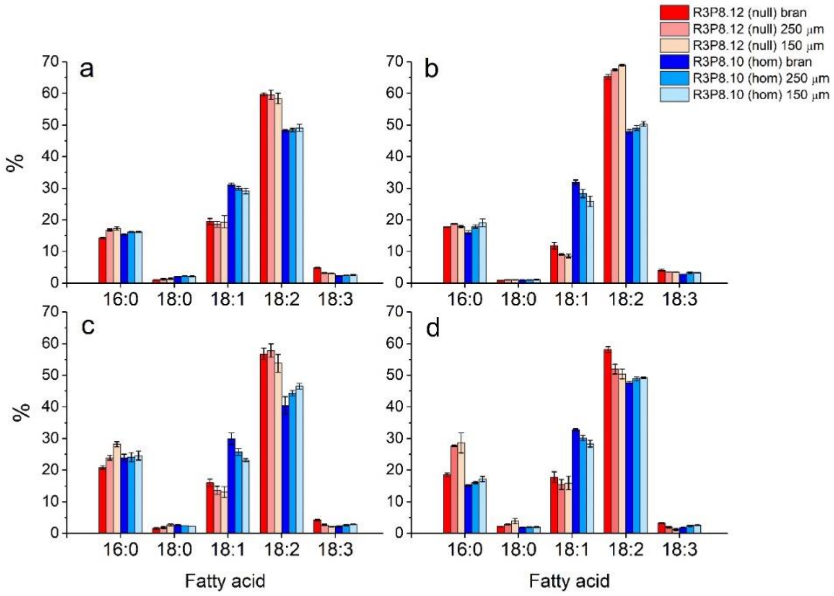

Figure S4. Fatty acid profiles of lipid classes in three different flour fractions (bran, 250 µm, and 150 µm mesh) from mature wheat grains from field grown AsWRI1-wheat (line R3P8.12, null and line R3P8.10, homozygous with 12 gene inserts). Profiles are given as percentage by weight of total major fatty acids (16:0 (palmitic acid), 18:0 (stearic acid), 18:1 (oleic acid), 18:2 (linoleic acid), and 18:3 (linolenic acid)). Results are mean values of three replicates (i.e. plots) ± standard deviation.
